# Non-Suicidal Self-Injury Among Adolescents: A Structural Model with Socioecological Connectedness, Bullying Victimization, and Depression

**DOI:** 10.1007/s10578-022-01319-6

**Published:** 2022-02-18

**Authors:** Ashley C. Baker, Jan L. Wallander, Marc N. Elliott, Mark A. Schuster

**Affiliations:** 1 Psychological Sciences and Health Sciences Research Institute, University of California, 5200 N. Lake Rd, Merced, CA 95343 USA; 2 RAND Corporation, Santa Monica, CA USA; 3 Kaiser Permanente Bernard J. Tyson School of Medicine, Pasadena, CA USA

**Keywords:** NSSI, Socioecological connectedness, Bullying victimization, Depressive symptoms, Adolescent

## Abstract

The objective was to examine the associations of socioecological connectedness with bullying victimization and depressive symptoms in early adolescence and with non-suicidal self-injury (NSSI) in mid-adolescence, and how these might differ between genders. Diverse adolescents (*N* = 4115; 49.1% girls) in the 7th grade reported on connections with parents/family, peers, school, and neighborhood, as well as bullying victimization and depressive symptoms, and NSSI in 10th grade (*M*^*e*^ = 16.1 years). Structural equation modeling with WSLMV indicated that the lower likelihood of NSSI in 10th grade was associated with higher perceptions of connections between adolescents and their families, both directly as well as indirectly through reduced bully victimization and depressive symptoms three years earlier. Higher school connectedness was indirectly associated with the lower likelihood of NSSI through bullying victimization and depressive symptoms. Paths to NSSI varied for girls and boys. Results advance the understanding of developmental pathways leading to NSSI in adolescent girls and boys.

## Introduction

Non-suicidal self-injury (NSSI), defined as direct and intentional self-inflicted bodily harm, including cutting behaviors but excluding suicidal intent and socially sanctioned forms of body modification [1], is a serious public health concern among adolescents. Prevalence estimates of past year NSSI in a non-clinical sample of US adolescents was almost 18%, with girls reporting double the prevalence of boys (23.8% vs 11.3%) [2]. This concerningly high prevalence rate suggests that NSSI is a major health concern among adolescent not only because of the direct tissue damage resulting from the injuries themselves, but NSSI has also routinely been associated with higher levels of depression and anxiety in youth [e.g., [3, 4]. Moreover, recent research suggests that NSSI in adolescence is linked to poorer mental health outcomes 10 years later [5]. Importantly, a systematic review of both cross-sectional and longitudinal studies concluded there are links between adolescent NSSI and both current and future suicidal thoughts and behaviors [6]*.* NSSI onset typically occurs between the ages 12 and 16 [7] and declines in later adolescence and young adulthood [2]. Therefore, the early- to mid-adolescent developmental period is a high-risk time for NSSI.

### Theoretical Framework for Socioecological Connectedness and NSSI

In this research we focus on socioecological connectedness. Although there is considerable variation in its operationalization across empirical studies, definitions tend to reference the subjective and/or structural features of social affiliation [e.g., [8]. Here we define it as a dynamic, multicomponent, complex factor that includes observable characteristics, such as embeddedness and social integration, as well as more subjective psychological experiences, such as closeness, belonging, caring, and supporting relationships [e.g., [9, 10]. Connectedness is rooted in numerous theoretical frameworks, including attachment theory [11] and ecological systems theories [12]. It is also a key component of the interpersonal theory of suicidal behavior [13], which suggests that thwarted belonginess (an indicant of low social connectedness) is associated with suicide risk, in part, due to the crucial psychological need of social belonginess not being adequately met. Connectedness may therefore function as a protective factor in adolescent health risk behaviors [14]. Indeed, the Centers for Disease Control and Prevention (CDC) identified connectedness as a potential target for interventions to reduce suicidal behaviors and thoughts, including NSSI among youth [15].

However, limited studies have explicitly explored how pathways between socioecological connectedness and NSSI differ for adolescent girls and boys, with mixed results. For example, one study found that connectedness with parents and a non-parental adult was associated with less NSSI, with the difference being more substantial in girls than boys [16]. Conversely, another study found that negative aspects of parenting, which overlaps with low parental connectedness, was associated with higher NSSI behaviors, particularly for boys [17]. Consequently, there are reasons to speculate that the paths from socioecological connectedness to NSSI may differ between girls and boys, but we cannot advance strong hypotheses regarding specific gender differences here due to the lack of consistent findings in this area.

### Levels of Socioecological Connectedness

Strong bonds with parents can support adolescent development. *Parent-family connectedness* is defined as the extent to which adolescents feel loved, cared for, valued, and respected by their parents and family [14]. Positive and healthy connections between adolescents and parents has been found to be a protective factor against NSSI engagement [e.g., [18, 19]. Furthermore, adolescents’ strong connections with prosocial peers can protect against a broad range of health risk behaviors [10]. *Peer connectedness* has been defined as perceptions of support, caring, and trust between adolescents and their peer groups [14]. Although limited work is available on peer connectedness and NSSI, one study found that the nomination of a best friend, indicating one salient aspect of connectedness with a peer, negatively predicted subsequent engagement in NSSI [20]. Conversely, another study found that when controlling for family, school, and other-adult connectedness, higher levels of peer connectedness was associated with an increased risk of NSSI [21].

Another salient social context in adolescence is the school environment. *School connectedness* reflects the adolescents’ sense of belonginess and bonding to one’s school [10]. Research has largely revealed school connectedness to be associated with reduced suicidal thoughts and behaviors [8, 22]*.* Among gay and lesbian high schoolers, school connectedness served as a particularly important protective factor against repetitive NSSI behaviors [18]. Yet one study that examined multiple domains of connectedness, did not find support for school connectedness on NSSI reduction [23]. Finally, *neighborhood and community connectedness* has been operationalized as sense of connection to and trust with others outside the more immediate social context of family and peers. Studies of connectedness to neighborhood and community and NSSI are sparse. One study found that caring relationships with non-parent adults was a protective factor for self-harm behaviors, including NSSI [24], whereas another did not find this association [23]. However, the scarcity of research like this has led to the call for more examination of how neighborhood and community connectedness might affect NSSI [8].

Taken together, these studies suggest the importance of social connectedness across multiple levels in reducing NSSI for adolescents, but more work concerning prospective predictors of NSSI would be of value. Whereas most research on NSSI has examined the role of interpersonal factors and psychopathology, research that examines the structural and subjective features of connectedness across contexts with particular attention to mechanisms is needed [21, 25]. Connectedness is preferably construed as a subjective experience, as defined for each level above [26]. However, in the absence of indications of perceived functional connectedness, knowledge about social structures that may engender connectedness can also be informative [27].

### Linking Socioecological Connectedness to NSSI Among Adolescents

The developmental transitions occurring in the period from early to mid-adolescence include significant social changes among others. There is the expansion of social connections to people outside the family and beyond the one or two friends that are more common prior to this period. However, this may challenge some, such that experiencing poor connectedness may increase certain problematic experiences among adolescents. We focus here on bullying victimization and depressive symptoms because both have consistently been associated with NSSI [e.g., [20, 28] and show an increased prevalence in early to mid-adolescence [e.g., [29, 30]. One of the reasons bullying victimization and depressive symptoms might be antecedents to NSSI is because both involve significant negative affect and adolescents who practice NSSI report that this reduces the intensity of negative affect [e.g., [31, 32]. Likewise, weak social connectedness among adolescents has also been associated with more bullying victimization [33, 34] and more depressive symptoms [e.g., [9, 10], suggesting potential mechanisms linking social connectedness with NSSI. Furthermore, more bullying victimization has been linked to NSSI through higher depressive symptoms [35, 36], but when exposed to positive parenting this association significantly decreased [36].

**Fig. 1:**
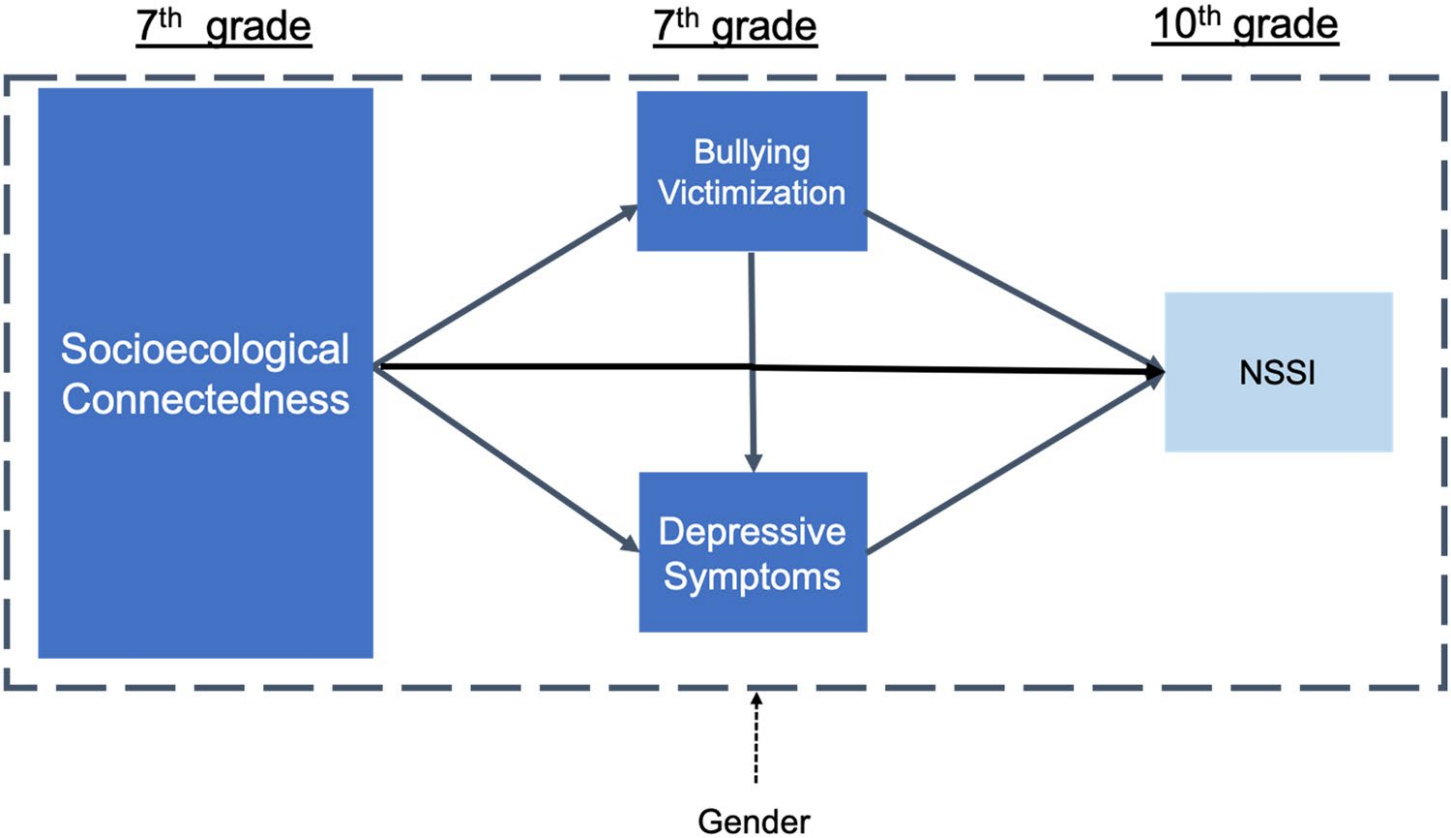
Conceptual hypothesized study model; NSSI = non-suicidal-self-injury

Given that the onset of depression typically occurs in early adolescence [37], and that bullying victimization is at the highest levels during middle school years [38], research is needed to inform how these experiences may precede NSSI when it most commonly occurs in mid-adolescence [2]. Based on these threads of empirical evidence, we propose a conceptual model, shown in Fig. 1, where social-ecological connectedness across parents and family, peer, school, and neighborhood levels in early adolescence are associated with a lower likelihood of NSSI in mid-adolescence, and poor connections are associated with higher likelihood of bullying victimization and depressive symptoms, which in turn are associated with a higher likelihood of NSSI.

### Study Aims and Hypotheses

Despite the significant health issues being associated with NSSI, we are still far from a coherent explanation of the occurrence of NSSI. There has been little research beyond identification of individual or smaller sets of risk factors. Theory-driven research is scant. We need to better understand psychosocial processes involved in the development of, or protection against, NSSI, especially in early to mid-adolescence when this reaches its peak prevalence. Theory and prior research suggest that socioecological connectedness provides a promising theoretical basis for understanding NSSI. Although individual socioecological connectedness factors are supported, simultaneous examination is absent into the role of the different levels of socioecological connectedness, leading to an incomplete understanding. Yet, low socioecological connectedness is likely not the most proximal influence on NSSI. Rather, poor connectedness may increase the development of problematic experiences in adolescence, such as bullying victimization and depressive symptoms, which have been strongly linked to increased likelihood of NSSI. However, we are not aware of any research examining such a cascading developmental conceptualization of NSSI.

Therefore, this study aimed to illuminate how the relationships among socioecological connectedness, bullying victimization, and depressive symptoms in early adolescence may illuminate the occurrence of NSSI subsequently in mid-adolescence. Prior research that has examined associations between multiple levels of socioecological connectedness and NSSI in adolescence has predominately been at one time point [e.g., [19, 21, 23]. However, measuring hypothesized contributing factors at the same time as NSSI can lead to biased or incomplete understanding. To improve on cross-sectional methods used in most previous research into the role of socioecological connectedness, the associations of interest here will be examined longitudinally, which enables consideration of the timing of potential influences and enhance our understanding of potential causal processes. Moreover, it remains unclear whether social connectedness operate the same for girls and boys. To address these several gaps in prior research, we examined three aims with associated hypotheses depicted in our conceptual model shown in Fig. 1.

1. To compare the prevalence of NSSI in mid-adolescence by gender. We expected (H1) that girls would report higher prevalence of NSSI compared to boys.
2. To test a model of longitudinal associations among adolescents’ perceptions of social connectedness in early adolescence and bully victimization, depressive symptoms, and NSSI in mid-adolescence as depicted in Fig. 1. Consequently, three hypotheses were tested: (H2) Each level—parent-family, peer, school, and neighborhood—of socioecological connectedness will be negatively associated with NSSI. (H3) Bullying victimization will be positively associated with depressive symptoms and NSSI, and in turn, depressive symptoms will be positively associated with NSSI. (H4) Perceptions of higher socioecological connectedness will be indirectly linked to reduced likelihood of NSSI behaviors through the reduced likelihood of bully victimization and lower depressive symptoms.
3. To explore how these association between distal and proximal predictors of NSSI (see Aim 2 and Fig. 1) differed between girls and boys. However, gender-differentiated hypotheses cannot be proposed at this time in the absence of guiding literature.

## Methods

Data came from *Healthy Passages™*, a multisite, longitudinal community cohort study of health-related behaviors and outcomes [39]. We utilized data from the 7th and 10th (2009–2011) grade assessments, defining early (ages 12–13) and mid- (ages 15–16) adolescence. Institutional review boards at each study site approved the study.

### Participants

Participants were recruited from public schools with ≥ 25 students enrolled in regular 5th grade classrooms in schools in and around metropolitan areas of Birmingham, Houston, and Los Angeles. A two-stage probability sampling procedure was used to select schools and students with school selection probabilities designed to attain similar proportions of (non-Latinx) Black, Latinx, and (non-Latinx) White participants. Sampling for the Healthy Passages study included 5^th^ graders in regular public-school classrooms in the three sites. Public schools within the three study site communities were randomly selected with probabilities proportionate to a weighted measure of the scarcity of a school’s students relative to race/ethnicity targets to ensure adequate sample sizes of the three largest racial/ethnic groups within the U.S. All 5th grade students within selected schools were invited to participate [39]. Among families who provided permission to be contacted and completed interviews in 5th grade (*N* = 5147; 2607 girls), 89% (*n* = 4289) completed both the 7th and 10th grade assessments. We analyzed data from the 4,115 (85% of original sample) adolescents who had parent data at 7th and 10th grade, resulting in a distribution that was very similar to the 5th grade sample regarding race/ethnicity and gender. Furthermore, those who did not complete 7th grade or 10th grade parent assessments were excluded from the analysis as this could compromise the assessment of parent-family connectedness. Those who were excluded from the analysis did not differ on any of the study variables compared to those who completed the assessments (details available from authors).

### Procedures

Two trained interviewers completed the full assessment protocol with the parent and adolescent either at their home or an alternative site. Assessments were administered with each individually in a private space using a computer-assisted personal interview method. A Spanish version, developed using standard back-translation methods, could be chosen by either at each assessment (selected at least in part at 5th grade: 8% of adolescents, 23% of parents; 7th grade: 4% of adolescents, 30% of parents; 10th grade: 30% of parents). The exception was for adolescents at 10th grade, at which time all were expected to be fluent in English after at least five years of U.S. education. The same procedures were repeated at each assessment.

### Measures

#### Non-Suicidal Self Injury

NSSI was self-reported at 10th grade using one item adopted from the state-level YRBS surveys [40], “During the past 12 months how many times did you do something to hurt or injure yourself on purpose without wanting to die, such as cutting, scraping, burning, or bruising yourself?”. Due to the skewed nature of responses on the original item, a dichotomous variable was created to contrast adolescents who had engaged in NSSI one or more times (1) in the past 12 months with those who had not (0).

#### Parent-Family Connectedness

The 13-item adapted scale from the Parent–Child Connectedness Questionnaire [41] was used to assess adolescent perceptions of connectedness with their parents and family at 7th grade. Items addressed perceptions of warmth, acceptance, closeness, and caring from mothers and fathers separately (e.g., “How close do you feel to {your mother}?”) on a 1 (*not at all*) to 5 (*very much*) scale. An average score across items was computed. For the current study, internal consistency was α = .86.

#### Peer Connectedness

Adolescents’ perception of connectedness with peers at 7th grade was measured with a single-item requesting the number of close or best friends, based on a similar approach in previous research [42, 43]. Response choices ranged from none (0) to 20 + friends (20). However, given the highly positively skewed data, a median split was used to create a dichotomous variable of less connected (≤ 4 = 0) vs more connected (> 4 = 1).

#### School Connectedness

Adolescents in 7th grade completed the ADD-Health school connectedness scale [41], consisting of five items (e.g., “You are happy to be at your school”) with response options ranging from *strongly disagree* (1) to *strongly agree* (4). An average score across all items was computed. For this study, internal consistency was α = .83.

#### Neighborhood Connectedness

Adolescents’ perceptions of connectedness with their neighborhood was measured at 7th grade using the Social Interaction Scale adapted from Sastry et al. [44]. This three-item scale assess adolescents’ perceptions of safety in their neighborhood and how many of their neighbors they know. (e.g., “How many of the kids in your neighborhood do you know? Would you say…?” *none* (1) to *most* (3)). An average score across the three items was computed, which had an internal consistency α = .50. Whereas this indicated a low internal consistency in absolute terms, it is based on only three items that cover heterogenous aspects of neighborhood connectedness that may not be strongly correlated.

#### Bullying Victimization

A single item of school-based bullying experiences was used at 7th grade [45], ‘‘How often have you been bullied in the past 12 months?’’ The five-point response scale ranged from *never *(1) to ‘‘*a few times a week* (5). Because the data were skewed, a binary variable was created to contrast adolescents who had been bullied at least once in the past year (1) with those who did not report being bullied (0). Test–retest reliability estimates have ranged κ = .60–.70 [46].

#### Depressive Symptoms

Adolescents’ depressive symptoms were measured in 7th grade with self-report using the Major Depressive Disorder DISC Predictive Scale, which has shown satisfactory reliability and validity for efficiently screening adolescents [47]. Five items ask youth about depressive symptoms (e.g., “Has there been a time when nothing was fun for you and you just were not interested in anything”) with response options *yes* or *no*. The number of yes responses (0–5) constituted a depressive score, which in this study had α = .66 and was comparable to a previous report [48].

#### Gender

Adolescents were asked to indicate which sex-based gender assigned at birth, girl (0) or boy (1), best described them.

#### Covariates

Adolescent age at 10th grade and highest level of education completed in the household (4 categories) were reported by the parent.

### Statistical Analysis

All analyses were conducted using weights in a manner that accounted for the complex survey design, including the effects of design, non-response, and attrition, clustering of youth within schools in each area, and stratification by site [39]. Consequently, weighted results reported here account for differential attrition over time and represent the population in the sampling frame of the three defined communities. First, using independent t-tests and chi-square analyses, we examined descriptive information to determine whether girls and boys differed on study variables. Next, we examined bivariate correlations among study variables in the full sample and then separately in girls and boys. Structural equation modeling via Mplus v.8.4 [49] was used to estimate the hypothesized path model (Fig. 1). Highest household education and age at 10th grade were controlled for on NSSI. Missing data was less than 4% on all variables. Weighted least squares means and variance adjusted (WSLMV) was chosen as the estimator for the dichotomous outcome NSSI. We initially estimated the model in the full sample to test hypotheses H2-H4. To address aim 3 regarding gender differences, we conducted multigroup invariance testing, but this analysis could not converge. Therefore, still to inform about gender differences, we tested the model separately for girls and boys [50].

We use an iterative process in achieving model results that converge. Therefore, we start with the most encompassing measurement model, using as many observations as available for each construct. This most complex SEM analysis did not converge when accounting for the complex survey design. We then simplify the measurement of variables in the model in a step-by-step fashion to achieve convergence. In the case of the present model, we found that variables that were substantially non-normally distributed needed to be transformed into dichotomous observed variables for the model to converge.

Because the literature recommends using more than one measure of fit, especially when categorical data are used [51], we did not reject good model fit based on an individual fit index, but rather considered the following indices holistically to interpret the results: the χ^2^ index, the root mean square error of approximation (RMSEA), the comparative fit index (CFI), Tucker Lewis Index (TLI), and the standardized root mean square residual (SRMR). We interpret CFI and TLI values of > .90 to indicate good model fit, 80–.90 to indicate acceptable fit, and < .80 to indicate poor fit; for RMSEA and SRMR, values of < .05 indicate close fit, .05–.08 indicate fair fit, and > .10 indicate poor fit [52–54]. We note, however, that these cut-off values may not be applicable to all structural equation models [see [50], p. 277], and some authors recommend different cut-off values be implemented [e.g., [55]. The fit indices are indeed impacted by elements of model complexity, missing data, and even sample size. Therefore, rather than using these rules as strict cut-points, we have opted to use them as guidelines to aid in interpreting model fit. We also carefully considered the substantive interpretation when retaining the final model.

## Results

### Preliminary Analyses

Descriptive information on study variables is reported in Table 1. Mean age was 13.1 (SD = .6) years in 7th grade and 16.1(SD = .6 years) in 10th grade, 49.1% were female, and 46.5% identified as Latinx, 30.4% as Black, and 23.1% as White. NSSI behaviors in the past 12 months was reported by 6.5%. Bivariate correlations in the full sample are shown in Table 2. Highlighting associations with NSSI here, higher perceptions of each level of socioecological connectedness were associated with the lower likelihood of NSSI behaviors (*r* = − .03 to − .10,). Being bullied (*r* = .08, χ^2^(1) = 29.00) and reporting higher levels of depressive symptoms (*r* = .09) in 7th grade were associated with reporting NSSI behavior in the past 12 months while in 10th grade. Correlations for the separate gender groups (see Appendices 1 and 2) were consistent with those found for the full sample, with the exception that perceptions of peer connectedness were not associated with NSSI behaviors for boys.

**Table 1:** Sample descriptives and gender differences for study variables

Categorical variables	Overall sample		Gender		Gender difference
	(*N* = 4115)		Girls (raw *n* = 2093)	Boys (raw *n* = 2022)	
	Raw *n*	Weighted %	Weighted %	Weighted %	χ^2^ (1)
*Highest household education*					
< High school graduate	539	17.2	18.4	16.1	
High school graduate/GED	897	23.7	24.1	23.4	
Some college or 2-year degree	1244	29.7	30.1	29.4	
≥ 4-year degree	1396	29.3	27.5	31.1	
*Peer connectedness*					6.50, *p* = .011
Low connectedness (≤ 4)	2350	51.0	59.5^a^	54.8^b^	
High connectedness (> 4)	1765	49.0	40.5^a^	45.2^b^	
*Victim of bullying*					8.25, *p* = .004
Yes	1269	31.6	30.3^a^	32.7^b^	
No	2846	68.4	69.7^a^	67.3^b^	
*NSSI behaviors*					21.83, *p* < .001
Yes	267	6.5	8.2^a^	4.8^b^	
No	3848	93.5	91.8^a^	95.2^b^	

Continuous variables	*M*	SE	*M*	SE	M	SE	*t* (115)
Parent-family connectedness	4.4	.01	4.4^**a**^	.18	4.4^**a**^	.15	− 1.10, *p* = .275
School connectedness	3.3	.57	3.3^**a**^	.01	3.2^**b**^	.01	**2.61, *p* = .001**
Neighborhood connectedness	2.4	.01	2.3^a^	.02	2.4^b^	.01	**− 3.72, *p* = .001**
Depressive symptoms	1.6	.03	1.7^**a**^	.05	1.5^**b**^	.02	**3.30, *p* < .001**

NSSI = non-suicidal self-injury; bold indicates significant gender difference

^a,b^Different letter superscript in a row represent significant differences between gender

**Table 2:** Correlation matrix of study variables for full sample

Model measures	1	2	3	4	5	6	7	8	9	10
1. Parent-family connectedness	1	–	–	–	–	–	–	–	–	–
2. Peer connectedness	**.04**	1	–	–	–	–	–	–	–	–
3. School connectedness	**.42**	**.08**	1	–	–	–	–	–	–	–
4. Neighborhood connectedness	**.19**	.02	.20	1	–	–	–	–	–	–
5. Victim of bullying	**− .12**	− .01	**− .13**	**− .06**	1	–	–	–	–	–
6. Depressive symptoms	**− .21**	− .01	**− .18**	**− .08**	**.22**	1	–	–	–	–
7. NSSI	**− .10**	**− .03**	**− .06**	**− .05**	**.08**	**.09**	1	–	–	–
8. Sex	.02	**− .05**	**− .05**	**.08**	**.04**	**− .04**	**− .07**	1	–	–
9. Highest household education	**.13**	**.01**	**.15**	**.07**	− .01	− .01	− .03	.03	1	–
10. Age at 10th grade	**− .08**	**− .04**	− .04	.03	.01	.02	.01	**.06**	**.05**	1

*NSSI* = non-suicidal self-injury; gender:  0 = females; 1 = males. Bolded correlations = *p* < .05

### Gender Differences for Study Variables

Gender differences for study variables are reported in Table 1. Girls reported being almost twice as likely to have engaged in NSSI in the past 12 months as boys (8.2% vs 4.8%). Highlighting other significant findings here, girls compared to boys reported lower perceptions of being connected to their school and neighborhood environments, less connected to friends, and more depressive symptoms, whereas boys more often than girls reported being bullied in the past year.

### Path Model for Total Sample

**Fig. 2:**
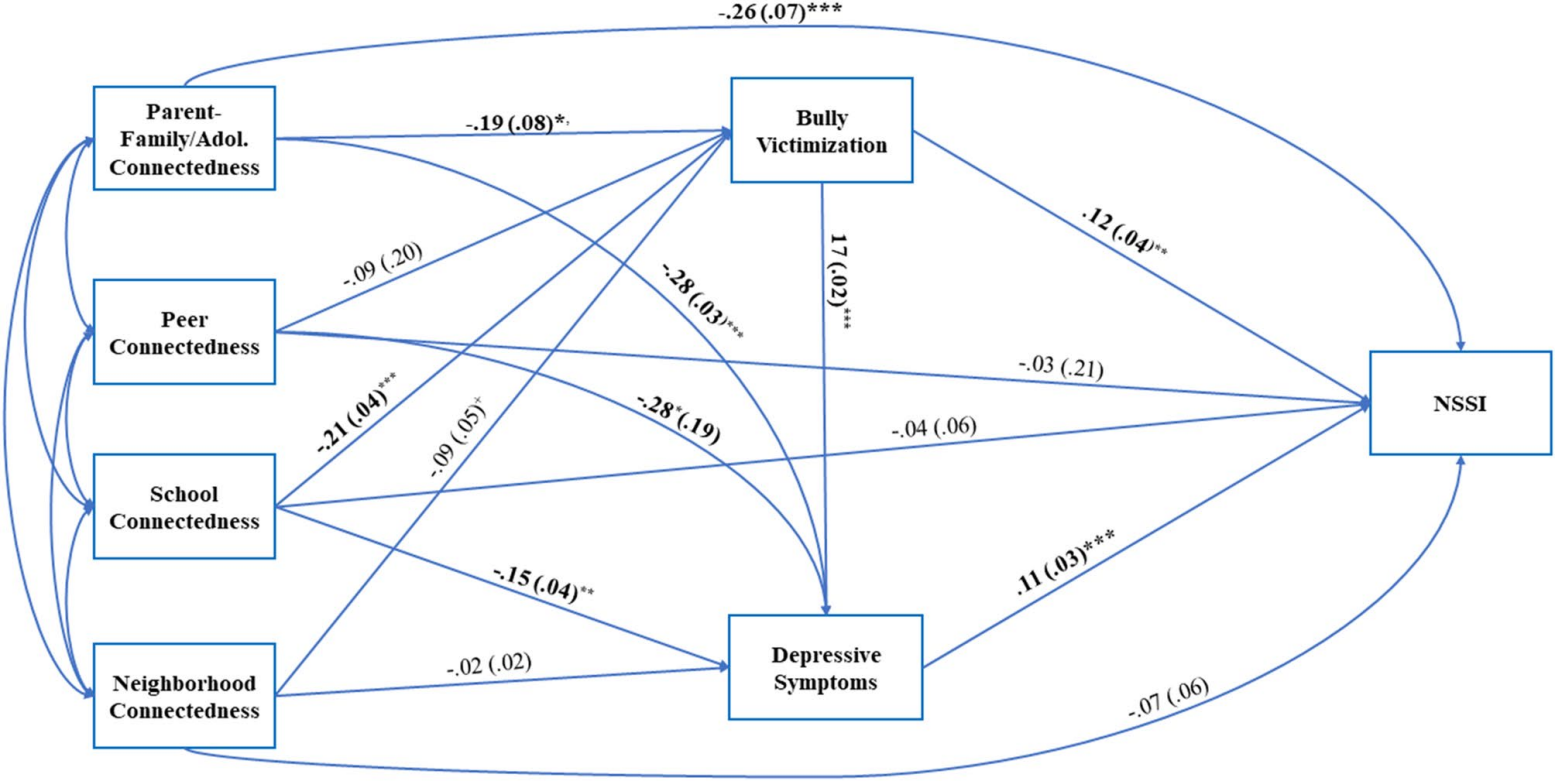
Unstandardized path coefficients (standard errors) for the total sample (*N* = 4115) structural model. Age at 10th grade and highest household education were controlled for on NSSI. Indirect path coefficients and fit indices are presented in Table 3. Bold indicates significant coefficients. NSSI = non-suicidal self-injury. *p < .05, **p < .01, ***p < .001

Path coefficients for the total sample model are shown in Fig. 2 (unstandardized) and Appendix Fig. 3 (standardized), and fit indices and indirect effects are reported in Table 3. The hypothesized model had good fit to the data across all fit indices. Results relevant to the hypotheses included: (1) Higher parent-family connectedness at 7th grade was associated with the lower likelihood of NSSI behaviors at 10th grade, whereas peer connectedness, school connectedness, and neighborhood connectedness were not; (2) higher parent-family connectedness and school connectedness were associated with the lower likelihood of bully victimization; (3) higher parent-family, peer, and school connectedness were associated with lower depressive symptoms; and (4) reporting being bullied and higher depressive symptoms were both positively associated with NSSI behaviors. In addition to these direct paths, seven indirect paths were supported, along with several additional indirect paths that approached significance (see Table 3). Of note, the indirect paths from parent-family connectedness and school connectedness to NSSI through bully victimization and/or depressive symptoms were significant. Paths from peer connectedness and neighborhood connectedness to NSSI through bully victimization and depressive symptoms were not significant.

**Table 3:** Path coefficients and fit indices for structural model for overall sample and gender

Paths	Total sample (*n* = 4115)			Girls (*n* = 2093)			Boys (*n* = 2022)		
	Coefficient (SE)^a^			Coefficient (SE)^a^			Coefficient (SE)^a^		
	Unstand	Stand	*p*	Unstand	Stand	*p*	Unstand	Stand	*p*
Parent-family connectedness to bully victimization to NSSI	− .03 **(**0.01)	− .01 (.04)	**.023**	− .02 (.02)	− .01 (.01)	.223	− .04 (.02)	− .02 (.01)	**.035**
Parent-family connectedness to depressive symptoms to NSSI	− .03 **(**.01)	− .02 (.01)	**.001**	− .05 (.02)	− .03 (.01)	**.002**	− .00 (.01)	− .00 (.01)	.764
Parent-family connectedness to bully victimization to depressive symptoms to NSSI	− .01 (.01)	− .00 (.00)	**.015**	− .01 (.00)	− .00 (.00)	**.021**	− .00 (v01)	− .00 (.00)	.763
Parent-family connectedness to NSSI (direct)	− .26 (.07)	− .14 (.04)	** < .001**	− .31 (.10)	− .16 (.05)	**.001**	− .17 (.12)	− .08 (.06)	.144
Parent-family connectedness to NSSI total effect	− .33 (.08)	− .17 (.04)	** < .001**	− .40 (.10)	− .20 (.05)	** < .001**	− .21 (.12)	− .10 (.06)	.070
Peer connectedness to bully victimization to NSSI	− .01 (.02)	− .01 (.00)	.226	− .02 (.04)	.00 (.00)	.873	− .00 (.06)	.02 (.01)	.106
Peer connectedness to depressive symptoms to NSSI	− .03 (.02)	− .00 (.00)	.121	− .04 (.04)	− .00 (.00)	.305	− .00 (.02)	− .00 (.00)	.774
Peer connectedness to bully victimization to depressive symptoms to NSSI	− .00 (.01)	.00 (.00)	.115	− .01 (.01)	.00 (.00)	.866	− .00 (.00)	− .00 (.00)	.762
Peer connectedness to NSSI (direct)	.03 (.21)	.03 (.03)	.405	.01 (.09)	.01 (.04)	.887	.13 (11)	.06 (.05)	.227
Peer connectedness to NSSI total effect	.06 (.06)	.03 (.03)	.356	.01 (.09)	.00 (.04)	.949	.17 (.11)	.08 (.05)	.137
School connectedness to bully victimization to NSSI	− .03 (.01)	− .01 (.01)	**.020**	− .02 (.02)	− .01 (.01)	.180	− .04 (.02)	− .02 (.01)	**.049**
School connectedness to depressive symptoms to NSSI	− .02 (.01)	− .01 (.00)	**.005**	− .03 (.01)	− .02 (.01)	**.008**	− .00 (.01)	− .00 (.00)	.759
School connectedness to bully victimization to depressive symptoms to NSSI	− .01 (.01)	− .00 (.00)	**.002**	− .01 (.00)	− .01 (.00)	**.006**	− .00 (.00)	− .00 (.00)	.766
School connectedness to NSSI (direct)	− .04 (.06)	− .03 (.03)	.461	− .06 (.08)	− .04 (.05)	.435	− .04 (.05)	− .03 (.06)	.558
School connectedness to NSSI total effect	− .10 (.06)	− .05 (.03)	.115	− .13 (.08)	− .07 (.05)	.134	− .10 (.11)	− .05 (.06)	.346
Neighborhood connectedness to bully victimization to NSSI	− .01 (.01)	− .01 (.00)	.126	− .00 (.01)	− .00 (.00)	.582	− .03 (.02)	− .01 (.01)	.116
Neighborhood connectedness to depressive symptoms to NSSI	− .00 (.01)	− .00 (.00)	.703	− .00 (.01)	.00 (.00)	.895	− .00 (.00)	− .00 (.00)	.811
Neighborhood connectedness to bully victimization to depressive symptoms to NSSI	− .00 (.01)	− .00 (.00)	.102	− .00 (.00)	− .00 (.00)	.515	− .00 (.00)	.00 (.00)	.775
Neighborhood connectedness to NSSI (direct)	− .06 (.07)	− .03 (.03)	.263	− .12 (.09)	− .06 (.04)	.174	− .05 (.15)	− .02 (.07)	.719
Neighborhood connectedness to NSSI total effect	− .09 (.07)	− .04 (.03)	.170	− .13 (.09)	− .06 (.04)	.159	.01 (.15)	.01 (.06)	.882
**Fit indices**									
*Χ*^*2*^*(df)* [*p*-value]	9.44 (8) [> .30]			1.90 (4) [> .74]				8.20 (4) [> .09]	
Comparative fit index (CFI)	.99			.99				.96	
Tucker-Lewis Index (TLI)	.95			.97				.84	
Root mean square error of approximation (RMSEA) [CI]	.01 [.00 to .03]			.01 [.00 to .04]				.02 [.00 to .04]	
Standardized root mean square residual (SRMR)	.07			.07				.09	

**Bold** = significant *p* values. SE = standard error of estimate, Unstand. = unstandardized coefficients, Stand. = standardized coefficients

^a^Coefficients have been rounded but retain sign from original value

### Path Models for Girls and Boys

**Fig. 3:**
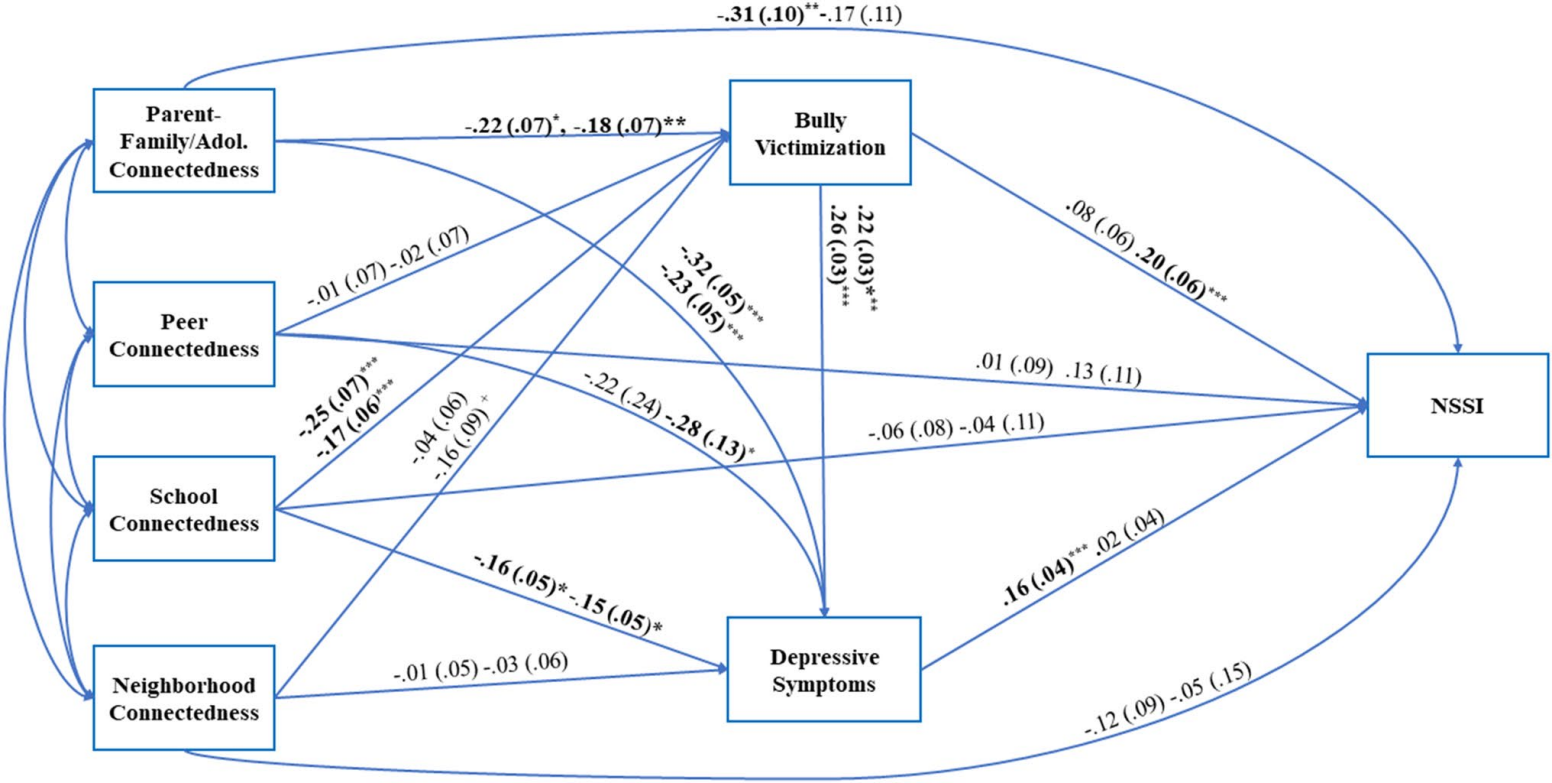
Unstandardized path coefficients (standard errors) for girls (*n* = 2093) and boys (n = 2022) structural model. The first coefficient for each path represents results for girls. Age at 10th grade and highest household education were controlled for on NSSI. Indirect path coefficients and fit indices are presented in Table 3. Bold indicates significant coefficients. *NSSI* non-suicidal self-injury. *p < .05, **p < .01, ***p < .001, ^+^*p* < .10

The model was tested separately for each gender group [50], which showed good fit among girls and adequate fit among boys (see Table 3). Path coefficients reported for girls in Fig. 3 (unstandardized) and Appendix Fig. 5 (standardized) largely mirrored those for the total sample with only two exceptions. Unlike in the total sample, for girls the path from peer connectedness to depressive symptoms was not significant, nor was the path from bullying victimization to NSSI. The same indirect paths were significant for girls as for the total sample.

In comparison, results for boys (see Fig. 3, Table 3, and Appendix Fig. 5) departed more from those for the total sample. For boys, none of the forms of socioecological connectedness was directly associated with NSSI. Higher school and neighborhood connectedness were associated with the lower likelihood of bullying victimization, and higher parent-family connectedness was associated with fewer depressive symptoms for boys. Consistent with the overall and girls sample, being bullied was associated with higher depressive symptoms for boys as well. In contrast with girls, being bullied was positively associated with NSSI behaviors for boys, whereas depressive symptoms were not. Indirect paths revealed associations for boys between parent-family connectedness and school connectedness with NSSI through being bullied.

## Discussion

This study aimed to advance our conceptual understanding of potential mechanisms involved in NSSI from early to mid-adolescence, and what differences there may be between boys and girls, given the much higher prevalence of NSSI among girls. Consistent with recent research on a non-clinical sample of adolescents [2] as well as our first hypothesis, girls in 10th grade reported almost twice the prevalence of NSSI behaviors in the past 12 months as boys, 8.2% versus 4.8%. Furthermore, the proposed conceptual model fit the data well for the overall sample. Results showed that the lower likelihood of NSSI behaviors in 10th grade was associated with perceptions of greater connectedness between adolescents and their families both directly as well as indirectly through bully victimization and depressive symptoms three years earlier, in 7th grade. Perceptions of greater connectedness with schools was only indirectly linked to the lower likelihood of NSSI through less bully victimization and fewer depressive symptoms. Therefore, bullying victimization and depressive symptoms appear as important mechanisms linking the family and school environments to NSSI behaviors in mid-adolescence, yet in different ways for girls and boys. These findings are unique in demonstrating that depressive symptoms are particularly important for understanding NSSI among girls and possibly less so for boys, whereas bully victimization appears of particular importance for boys and less so for girls.

### Path Model for Total Sample

Results supported that for all adolescents, perceptions of greater connectedness with their parents and family during early adolescence were associated with the lower likelihood of NSSI behaviors three years later, partially supporting hypothesis two. This is in line with past theoretical and empirical work suggesting that for adolescents, feeling connected to parent-family environments that are characterized as warm, loving, and accepting likely plays the most important role in preventing NSSI behaviors [e.g., [9, 10, 14, 19]. Whereas we had hypothesized that adolescents’ perception of school connectedness would be directly associated with NSSI behaviors, results did not support that notion. The lack of direct effects of school connectedness on NSSI is consistent with other studies that also accounted for multiple connectedness contexts and self-harm behaviors [23, 24], which may point to the interactions of these contexts that are often unexplored in connectedness studies. It is also possible the observed variability in results linking NSSI and different forms of connectedness may be a consequence of latent factors influencing the data. In general, however, multiple lines of research converge to suggest that positive parent-family connectedness is the most salient factor of reduced self-harm behaviors followed by school connectedness [24].

Also, our results are consistent with past research that has generally showed less support for the positive effects of peer and neighborhood connectedness on NSSI [e.g., [23]. One explanation may be that our peer connectedness measure addressed only the structural component of connectedness, when other research has found that the quality of the relationship matters [56]. Additionally, some research suggests that knowing a friend who engages in the behavior may exert the most influence on self-harm behaviors [e.g., [20, 56], suggesting that we still have much to learn about the different ways in which connections with peers influence engagement in NSSI. Neighborhood and community connectedness may be most beneficial for certain groups of adolescents not examined here. For example, one study found that positive connections with tribal elders among US indigenous youth was a protective factor against suicidal thoughts and behaviors [56].

Along with showing a direct effect on NSSI for parent-family connectedness, there were also indirect paths through bullying victimization and depressive symptoms. Likewise, school connectedness was indirectly associated with NSSI through bullying victimization and depressive symptoms. These findings are in line with an ecological systems theory of health risk behaviors in adolescents [12], which emphasizes that adolescent health risk behaviors are situated between individual (e.g., depressive symptoms) and interpersonal (e.g., family, school, bullying victimization) factors. The results also suggest that emotional processes, such as depressive symptoms, should be considered in theoretical frameworks of NSSI [13]. To the best of our knowledge, this is the first study that has specifically examined with longitudinal data an integrated model of the role of bullying victimization and depressive symptoms between multiple forms of social connections in early adolescence and NSSI in mid-adolescence. A caveat must be raised, however, that connectedness, bullying, and depressive symptoms were measured concurrently in 7th grade, and therefore it is impossible to determine which preceded which. For example, whereas it is reasonable to hypothesize, as we have done here, that lack of social connectedness can precede bullying, it is also plausible that bullying leads to more depressive symptoms, which can result in a lack of social connectedness. Future prospective research on adolescent NSSI should consider incorporating these prominent psychological and social processes in a design that can better examine the timing of possible influences [57].

### Differences Between Girls and Boys

Yet, the results also demonstrated differences for boys and girls regarding pathways to NSSI. For girls, parent-family connectedness and depressive symptoms were important direct predictors of NSSI. In addition, depressive symptoms linked parent and family and school connectedness to NSSI for girls but not in boys. Past research has found that family environments characterized by low support (an element of low connectedness) were linked to more NSSI behaviors in adolescent girls through higher emotion dysregulation problems [58]. Given that NSSI typically occurs in the context of emotional distress [59], and that adolescent girls are at higher risk for depressive symptoms [60], our findings further add to the research that that NSSI is at least partially dependent on social contexts and emotional processes for girls.

For boys, being bullied in the 7th grade was the only significant direct predictor of NSSI three years later. Additionally, bullying victimization formed an indirect link from parent and family and school connectedness and NSSI, suggesting that being a victim of traditional bullying may be of particular importance for understanding NSSI in boys. A cross-sectional study reported that boys who were victims of bullying were more likely to self-harm compared to girls [61]. Speculatively, the process from boys’ connectedness with parents and school environments to NSSI may unfold through bullying victimization, in part because boys are less likely than girls to disclose bullying victimization to parents and teachers [e.g., [62, 63]. Thus, NSSI behavior could function as an extreme means of non-verbal communication [63]. Given that these are preliminary results, more research is needed to better understand how bullying victimization may link socioecological experience of boys and NSSI.

### Limitations and Future Directions

NSSI, bullying victimization, and peer connectedness were measured using single items, which may result in reduced reliability. Future research can benefit from measuring variables with multiple items or validated scales. Measures of peer and neighborhood connectedness reflected structural rather than functional aspects of connectedness, which would have been preferable. Furthermore, we assessed only adolescent perceptions of connectedness with people and their environments, yet past research has shown that in the context of NSSI, adolescents and parents differ in their perceptions of family functioning [64]. Thus, future research should include multi-informant reports. Likewise, because depression is a complex and heterogenous construct, a richer assessment with better psychometric properties would have been advantageous. We were unable to examine transgender youth, who are most at-risk for NSSI behaviors [e.g., [18, 65] and should be addressed in future studies. Population studies should attempt to measure gender identity as a more fluid concept rather than just dichotomously. This should also be supplemented with research targeting transgender youth directly.

Additionally, because we were not able to investigate mediational paths and change over time, which require measurement across three time periods, the present finding should be considered a starting point for future research to examine these associations further. Repeated measurement of all variables over multiple occasions during development would be preferable. The low NSSI prevalence estimates reported here compared to previous studies likely are due to the non-anonymous, albeit confidential, personally intimate assessment method employed. Research into sensitive behaviors, such as NSSI, may require anonymous, group-based survey assessment. Finally, alternative analysis approaches may yield valuable insights into latent profiles that inform about potential influences on NSSI, such as through latent class analysis or growth mixture modelling.

### Implications

Once replicated, these findings may guide family and school-level interventions in reducing NSSI, focusing on reducing depressive symptoms for girls and bullying victimization for boys. Reviews of interventions for self-harm behaviors including NSSI have found that the most efficacious interventions focused on improving adolescents’ relationships with parents and family [66, 67]. Our results suggest that strengthening parent-family connectedness, such as through positive parent practices, may reduce NSSI [68, 69]. On the other hand, schools have the unique ability to reach the vast majority of youth. Additionally, we are not aware of a NSSI-specific prevention or intervention program in schools that focuses on promoting connectedness. However, *Sources of Strength*, a school-based and peer-led suicide prevention approach builds on naturally occurring socioecological protective factors [70], has promising results in increasing youth-adult connections and emotion-regulation strategies and reducing suicide attempts [71]. It is feasible then, that given NSSI has been identified as a unique predictor of suicidal behaviors [72] and that NSSI and suicidal behaviors share many of the same risk factors, *Sources of Strength* could be adapted to include an NSSI-specific module. Finally, health professionals need routinely to screen for NSSI behaviors, bullying, and depressive symptoms, and inquire about adolescents’ family relationships and school experiences as part of their health assessments.

## Summary

Being embedded in high-quality close relationships and feeling socially connected to the people in one’s life are associated with decreased risk for a range of poor health outcomes. This study investigated associations of socioecological connectedness with bullying victimization and depressive symptoms in early adolescence and with subsequent NSSI in mid-adolescence among girls and boys using a sample of over 4000 adolescents from diverse backgrounds. First, in line with past research mid-adolescent girls report almost two times the engagement in past-year NSSI compared to boys. Second, the lower likelihood of NSSI occurred in the context of strong parent and family relationships for all adolescents. Contrary to our predictions, peer, school, and neighborhood connectedness were not directly associated with NSSI; however, we do not take this to mean that these contexts are unimportant, rather that healthy family environment is likely the most critical aspect of connectedness in adolescent NSSI behaviors. Bullying victimization and depressive symptoms appear to be pathways that link parent-family and school connectedness to NSSI. However, paths from various forms of social connections varied between girls and boys, specifically depressive symptoms seemed particularly important for girls, whereas bullying victimization was for boys. Speculatively, girls and boys may use NSSI for different functions in response to stressful experiences, like depressive symptoms and bullying victimization when connections with their parents and schools are weak. More inquiries to confirm these propositions are needed. Our findings further reinforce the potential for clinicians to contribute by inquiring about adolescent social relationships, bullying victimization, and depressive symptoms, and to endeavor to find ways to strengthen connections between adolescents and their families.

## Figures and Tables

**Fig. 4:**
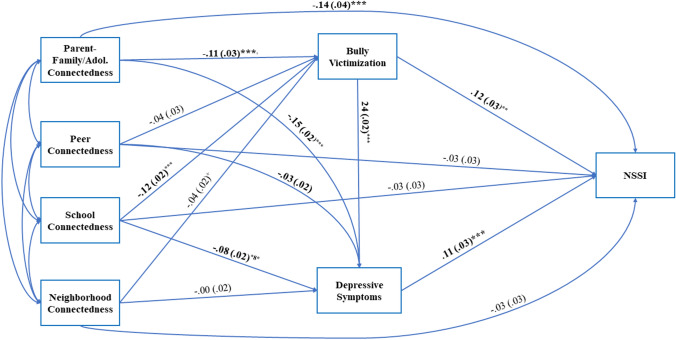
Standardized path coefficients (standard errors) for the total sample (*N* = 4115) structural model. Age at 10th grade and highest household education were controlled for on NSSI. Indirect path coefficients and fit indices are presented in Table 3. Bold indicates significant coefficients. *NSSI* non-suicidal self-injury. *p < .05, **p < .01, ***p < .001

**Fig. 5:**
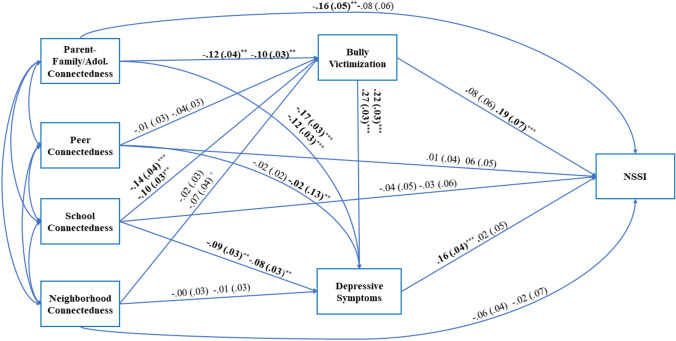
Standardized path coefficients (standard errors) for girls (*n* = 2093) and boys (*n* = 2022) structural model. The first coefficient for each path represents results for girls. Age at 10th grade and highest household education were controlled for on NSSI. Indirect path coefficients and fit indices are presented in Table 3. Bold indicates significant coefficients. *NSSI* non-suicidal self-injury. *p < .05, **p < .01, ***p < .001, ^+^*p* < .10

## Appendix

See (Table 4 and Figs. 4,5).

**Table 4:** Correlation matrix of study variables for girls and boys samples

Boys (raw n = 2022; below diagonal)	Girls (raw *n* = 2093; above diagonal)								
	1	2	3	4	5	6	7	8	9
1. Parent-family connectedness	1	.03	**.44**	**.19**	**− .15**	**− .26**	**− .12**	**.11**	**− .12**
2. Peer connectedness	**.05**	1	.04	.01	**− .03**	**− .05**	**− .05**	.03	.01
3. School connectedness	**.42**	**.12**	1	**.19**	**− .14**	**− .22**	**− .10**	**.16**	**− .06**
4. Neighborhood connectedness	**.19**	.04	**.22**	1	**− .07**	**− .09**	**− .07**	**.05**	− .01
5. Victim of bullying	**− .12**	− .02	**− .11**	**− .07**	1	**.21**	**.11**	− .03	.03
6. Depressive symptoms	**− .18**	− .01	**− .19**	**− .08**	**.23**	1	**.12**	− .04	**.05**
7. NSSI	**− .06**	**− **.03	**− .03**	− .01	**.04**	**.12**	1	− .01	− .01
8. Highest household education^*^	**.16**	**− .07**	**.15**	**.09**	.02	− .01	**− .05**	1	**− .16**
9. Age at 10th grade^*^	− .04	− .01	− .02	**.05**	− .03	.001	.02	**− .09**	1

NSSI = non-suicidal self-injury; gender:  0 = females; 1 = males. Bolded correlations = *p* < .05

*Control variables in multivariate models
